# Exploring the Barriers and Facilitators Experienced by Palliative Health Care Providers Working with Patients Experiencing Homelessness during the COVID-19 Pandemic

**DOI:** 10.1089/pmr.2022.0051

**Published:** 2023-01-17

**Authors:** Claire Rollans, Justine Baek, Gary Bloch, Joyce Nyhof-Young, Trevor Morey, Naheed Dosani, Donna Spaner

**Affiliations:** ^1^ Department of Family and Community Medicine, St. Michael's Hospital, Toronto, Ontario, Canada.; ^2^ Department of Family and Community Medicine, University of Toronto, Toronto, Ontario, Canada.; ^3^ Primary Care, Inner City Health Associates, Toronto, Ontario, Canada.; ^4^ Academics Program, Women's College Hospital, Toronto, Ontario, Canada.; ^5^ Palliative Education and Care for the Homeless, Inner City Health Associates, Toronto, Ontario, Canada.; ^6^ Palliative Care, Toronto Grace Health Centre, Toronto, Ontario, Canada.

**Keywords:** COVID-19, health care providers, homeless, interviews, palliative care, underserved populations

## Abstract

**Background:**

Patients experiencing homelessness not only have higher rates of medical complexity, comorbidity, and mortality, but also face barriers to accessing palliative care services. In structurally vulnerable populations with palliative care needs, these barriers are compounded, creating significant challenges for both patients and providers that have important health equity implications.

**Objective:**

The aim is to explore the experiences of palliative care providers working with patients experiencing homelessness during the COVID-19 pandemic and understand the barriers they faced in providing care, as well as facilitators that aided in the success of their teams.

**Methods:**

Seven health care providers from two Canadian palliative outreach teams involved in delivering palliative care services to patients experiencing homelessness during the COVID-19 pandemic participated in audio-recorded and transcribed videoconferencing interviews. Analysis was completed using generic descriptive thematic analysis.

**Results:**

Five key themes were identified: (1) factors negatively impacting patient health, (2) use of technology, (3) care provider emotions, (4) care provider education and advocacy, and (5) outreach team factors.

**Conclusion:**

Identified barriers during the pandemic included worsening of existing patient vulnerabilities, as well as challenges incorporating technology into care. Providers faced increased emotional burden, with a rise in workload, stress, fear, and grief. However, several facilitators allowed teams to provide high-quality care to this vulnerable population, including team support, interprofessional collaboration, and advocacy and education initiatives. The outreach model also proved to be a highly flexible, resilient, and adaptable model for providing care during the COVID-19 pandemic.

## Background

Individuals experiencing homelessness face higher rates of medical complexity, comorbidity, and mortality, and numerous barriers to accessing palliative care services.^1^ Barriers include unstable living environments, lack of social services, negative experiences with health care providers, and stigma in the health care system.^1–4^ Comorbid mental illness or substance use disorder further impede access to palliative care programs.^1–4^ Most mainstream palliative care services are not designed with these barriers in mind, often relying on a safe living space and an engaged support system to allow clients to die in place.^3,4^ Furthermore, many medical practitioners have limited education about patients experiencing homelessness, including harm reduction and trauma-informed care.^3–5^ Shelter workers are often equally ill-equipped to support terminally ill patients, given their lack of training and insufficient staffing.^4,5^

In response, for the past few years several Canadian groups have created palliative care outreach programs tailored to the needs of individuals experiencing homelessness. Outreach programs refer to models of care delivery that focus on facilitating access to medical services and connection to social supports in settings where patients live or seek services in the community.^6^ Outreach teams are typically multidisciplinary, involving professionals such as nurses, social workers, and physicians working together to provide for the complex needs of patients who have difficulty engaging with traditional ambulatory or home-based models of care6. Through these programs, care can be provided in shelters, at drop in, or on the street when needed to meet the patient where they are.

The COVID-19 pandemic deepened existing barriers to equitable health care and created new challenges that impacted vulnerable communities at higher risk of infection with, and severe outcomes from COVID-19.^7^ At the peak of COVID-19, those with recent history of homelessness in Ontario were >20 times more likely to be admitted to hospital for COVID-19, >10 times more likely to require intensive care for COVID-19, and >5 times more likely to die within 21 days of their first positive test result.^8^ Crowding, high turnover, and lack of quarantine spaces and staffing in shelters presented challenges to outbreak control.^7,9^ Many community services for people experiencing homelessness closed.^7^ Palliative care workers also faced significant challenges due to inadequate resources and lack of personal protective equipment.^10^

This study is the first to explore the unique challenges facing palliative care providers of patients experiencing homelessness during the COVID-19 pandemic. The aim is to gain insights into the care barriers identified and factors helping teams navigate challenges in providing equitable care. These findings can then be applied by other health care teams working with marginalized communities to improve care delivery.

## Methods

### Setting and participants

For this qualitative study, purposive sampling of participants was conducted at two Canadian outreach teams serving the palliative care needs of marginally housed populations. Sampling was conducted on a rolling basis until thematic saturation was reached. The teams were based in two different Canadian provinces, with one team approximately twice as large as the other. Both teams included a similar mix of health professions, including social work, nursing, and physicians. All participants delivered palliative care services to patients experiencing homelessness during the COVID-19 pandemic. Ethics approval for the study was obtained through the Unity Health Research Ethics Board.

To be included in the study, participants had to

- be health care workers who spent at least one half-day per week providing palliative care to people experiencing homelessness for a minimum of six months during the COVID-19 pandemic,
- work at one of two identified Canadian study sites, and
- be conversant in English.

### Data collection

The 20- to 40-minute semistructured interviews were conducted over a secure videoconferencing platform during October and November 2021. Verbal consent was obtained before each interview. A semistructured interview guide (Supplementary Appendix S1) explored facilitators, clinical and organizational barriers, and health behaviors affecting delivery of palliative care during COVID-19. Minor iterative modifications were made to the guide as interviews progressed. Interview audio was digitally recorded, transcribed, and transcripts de-identified. Audio files were destroyed post-transcription.

### Data analysis

Interviews were analyzed using descriptive thematic analysis.^11^ All analysis was completed by coinvestigators with guidance from an experienced qualitative researcher. Data were coded using an iterative emergent approach whereby themes were developed from the data rather than existing theory. The data were coded independently by the two coinvestigators, and differences resolved through discussion.

Coded units were grouped into broader classifications based on meaning, with identification of domains, themes, and patterns based on the primary research question. New findings were checked and compared with earlier data in a constant comparison process.^12^

## Results

In total, seven participants completed interviews. They included four physicians, two nurses, and one social worker. Five themes were developed (Supplementary Table S1).

### Theme 1: Factors negatively impacting patient health

Without access to stable housing, respondents noted that patients were at higher risk of contracting COVID-19 in shelters and suffering severe complications. They reported shelter hotel programs, established by many jurisdictions as a response to the pandemic, offered temporary patient respite. However, displacement to hotels had unexpected negative outcomes, including increased social isolation and overdose deaths.

Mismatches between health care resources and policies and population care needs were seen to discourage patients from accessing care. Examples included lack of harm reduction supports in palliative care facilities such as safe opioid supply programs, lack of health insurance, lack of recognition of patients' street families, and visitor access.

Respondents stated that COVID-19 threatened an already fragile social safety net for these patients through cessation of community services such as drop-in centers, harm reduction services, and health care teams.

### Theme 2: Use of technology

Respondents identified that increased technology use in health care during COVID-19 created barriers for patients lacking access to communication devices. In response, teams distributed phones and tablets for patients to attend virtual visits with specialists, which were often obtained through philanthropic endeavors. The challenges of fostering therapeutic relationships virtually were noted; some patients were unable to engage with virtual platforms due to mental illness, disabilities, trauma, and so on. One team implemented a system of evaluating patients' medical stability and familiarity with communication devices to determine whether virtual care was appropriate.

Despite these difficulties, when virtual care was equitably accessible, it reduced travel barriers and increased volumes of patients seen, offering alternative easier ways for patients to access health care.

### Theme 3: Provider emotions

Respondents experienced increased pandemic-related emotional demands, including fear of COVID-19, grief, burnout, and moral distress, particularly in the context of increased patient volumes, staff shortages, multiple unexpected deaths, and overlapping health crises. They developed various coping strategies to maintain wellness and resiliency, including team debriefs and grief circles.

### Theme 4: Care provider education and advocacy

Sharing expertise with other providers improved competency in equitable palliative care approaches and delivery, as did grief workshops and grief circles with providers and use of tools for advance care planning during COVID-19. Advocating on behalf of their patients to shelters, COVID hotels, and inpatient health care teams was seen as key for moving patients into safer settings. These providers also advocated to their provincial governments to improve health policies, including prioritizing shelter residents for COVID vaccines. Increasing public awareness of their patients' needs by sharing personal narrative experiences became a useful advocacy strategy through op-eds, interviews, and other media.

### Theme 5: Outreach team factors

Key features of the outreach model of care that facilitated ongoing delivery of palliative care to participants' patients included working in multiple environments and settings with patients, the interprofessional and diverse skillsets of team members, and a shared commitment to continuing to provide in-person care.

These care providers also described working with their teams to develop new strategies for care and ways of supporting the work of other medical and social teams. In this way, the teams expanded their scopes of practice during the pandemic, while creating new partnerships with charities, volunteer organizations, community organizations, and medical staff. As one participant explained, “It did bring a lot of people together. It gave us a common enemy: COVID.”

Overall, participants described the outreach team model as flexible and resilient throughout the pandemic. This model was key to their success in maintaining high-quality patient care.

## Discussion

Outreach team members witnessed firsthand the barriers faced by persons experiencing homelessness and needing palliative care services during the COVID-19 pandemic. These health care providers highlighted preexisting barriers to care identified in previous systematic reviews, including lack of harm-reduction approaches in standard palliative care, and failure to recognize street families.^1,4,5^ The tenuousness of social safety nets patients rely on for daily living and how quickly services disappeared during the pandemic were also concerns.

Negative outcomes included reduced services, lower patient well-being, increased overdose deaths, social isolation, and displacement to unfamiliar areas of the city. These findings are consistent with previous study showing a disproportionate impact of pandemics on structurally vulnerable populations,^7^ with arguably a greater impact on palliative care patients who live with a high level of medical complexity and disability. Our results suggest that COVID-19 had a disproportionately negative effect on the health of palliative care patients experiencing homelessness and highlights the need for increased medical and social supports to help address the complex needs of this population.

Increased use of technology in health care delivery became the norm during the pandemic. Studies have found benefits to virtual palliative care, including expanding access to, and increasing the scale of, services, allowing participants to view facial expressions without masks, and reducing risk of disease transmission.^13,14^ However, the risk of poor connection, overuse in improper situations, and lack of accessibility for marginalized patients were also noted.^14,15^ This was borne out in our study; many patients lacked phone or computer access. To overcome this barrier, both teams worked with philanthropic organizations to provide phones or tablets. Increasing technological access did have benefits, including reducing patient travel barriers, facilitating specialist access, and increasing patient visit volumes. Our results suggest that increasing equitable access to technology for some palliative care patients experiencing homelessness may reduce barriers to palliative care service access.

Our results also highlight that virtual care is not a viable option for all patients. Patients with some mental health conditions, cognitive impairments, or sensory disabilities may be unable to effectively engage with virtual care.^14^ Providers also noted the importance of making in-person patient connections. As many patients in this population had previous negative experiences with the health care system, building trust through in person encounters was essential. Therefore, although increased access to technology can be beneficial, in-person care remains vital to quality palliative care provision for patients experiencing homelessness and must continue during public health crises.

The COVID-19 pandemic caused increased health care team strain through higher volumes and greater demands to fill care gaps. Participants expressed uncertainty about how the pandemic would progress and fear of getting or transmitting COVID-19. This echoes the emotional burden carried by home care workers,^16^ long-term care workers,^10^ and palliative care teams during the pandemic.^17,18^ However, as providers working with marginalized populations, our participants additionally reported moral distress from knowing the disproportionate effect the pandemic would have on their patients, as well as overlapping grief with unexpected deaths occurring due to COVID-19 and opioid overdoses. These results suggest a need for effective strategies to recognize and support health care providers experiencing moral distress during crises, particularly those working with marginalized populations.

With increased emotional demands from the pandemic, an important facilitator of team resilience was strong collegial support and shared team values, as noted by Cheng and Sin^17^ for palliative teams. Many of our providers spoke about their team connections, the importance of reflection and debriefing, and their shared commitment to equitable care provision. Although these coping methods were utilized before the pandemic, they became prioritized with the increased emotional strain of providing care during the pandemic. Our results strengthen the argument for developing strong support systems within health care teams to promote increased personal and team resilience during a crisis.

Participants also found new opportunities for advocacy and education. A major barrier to care access is the lack of palliative care training among shelter staff, along with a lack of knowledge about caring for marginalized populations among medical staff.^1,2,5,10^ Some palliative care teams turned to education to help expand care.^4,13^ Indeed, in our study, one team created an advance care planning tool for social service providers to help ensure patient agency in directing their goals of care. Providers also amplified their clients' voices to increase public awareness of the issues facing their patients experiencing homelessness through social media. Outreach teams have expert knowledge of their patients' palliative care experiences and are well positioned to act as health advocates and educators to improve their care.

Importantly, this study highlights the value of outreach care models in providing palliative care to patients experiencing homelessness during the COVID-19 pandemic. Outreach and shelter-based models of palliative care have been previously suggested as beneficial models of care for this population due to their relatively low cost, patient-centeredness, and ability to meet patients “where they are.”^1,4,12,19^ In this study, the flexibility of the outreach model allowed care teams to quickly pivot in response to new regulations. They did not stop or significantly alter their practices.

Therefore, outreach teams became a major resource, not only to patients but also to other care teams; they were relied upon to help facilitate visits with other providers, coordinate care, and fill in gaps for medical care and social needs. These teams are interdisciplinary by nature; this allowed them to problem-solve a multitude of complex needs within the changing pandemic landscape. The COVID-19 pandemic highlighted the critical role of these teams in maintaining care for vulnerable patients during times of crisis and demonstrated how outreach models could provide an even larger scope of care, if given the opportunity.

Several study limitations exist, as do opportunities for future research on this topic. First, we were only able to engage with members of two Canadian palliative outreach teams, but interdisciplinary data saturation was achieved. The experiences and insights of other similar teams in Canada and internationally may differ, but our lessons learned are likely to be applicable in other contexts. In addition, our scope was limited to provider experiences; patient perspectives would be highly valuable additions to future research.

## Conclusion

This article explores the experiences of interdisciplinary palliative care providers working with populations experiencing homelessness during the COVID-19 pandemic and offers insights into the unique barriers faced by this patient population. It also highlights facilitators that allowed these teams to remain resilient during this global health crisis, including increasing access to technology while maintaining in-person care, a supportive team environment, engagement in advocacy and education initiatives, and a collaborative interprofessional network, all within a flexible outreach model. As we move beyond the context of the COVID-19 pandemic, these insights can continue to be applied by other health care providers working with socially marginalized populations to build team priorities and improve care delivery.

## Supplementary Material

Supplemental data

Supplemental data

## Supplementary Material

Supplementary Table S1

Supplementary Appendix SA1
