# Supplementary material for: Exploring the Barriers and Facilitators Experienced by Palliative Health Care Providers Working with Patients Experiencing Homelessness during the COVID-19 Pandemic
Source: Palliat Med Rep. 2023 Jan 17;4(1):3–8. doi: 10.1089/pmr.2022.0051 (PMC9892914; doi:10.1089/pmr.2022.0051)
Supplement: Supplemental data [file Suppl_AppendixSA1.docx]

**APPENDICES**

**APPENDIX 1: Interview Guide**

Before the interview begins, the interviewer will provide the following points:

1. Identify that the interview is voluntary.
2. Participants do not have to answer all of the questions and may skip any question they do not wish to answer.
3. The discussion may include sensitive topics and some questions may be uncomfortable to answer.
4. They can pause or stop the interview at any time, for any reason.
5. The expected length of the discussion is approximately 30 minutes.
6. Remind participants that this session will be audio recorded.
7. Review that consent is implied by participating in the interview after verbal or written consent has been obtained.
8. Direct quotes may be used in future publications but will be de-identified.

Section 1: Introductions

1. Tell us about the Palliative Care Team setting in which you work.
2. What is your role on the team?
3. Who are the other team members and how do you work together?
4. What specific patient population do you work in?
5. In what settings/environments do you do your work?

Section 2: Changes during the pandemic

1. In what ways did your practice change at the beginning of the pandemic?

Probing questions:

- 1. What were the initial reactions from the team or from your patients?
  2. What were your biggest concerns at that time?
  3. What were some of the first changes you made and what were the effects of those changes?
  4. What were some of the first difficulties you encountered as a team? How did you deal with those difficulties?

1. How did your practice continue to change across the pandemic?

Probing questions:

- 1. What changes did you make?
  2. What did you learn from those changes?
  3. How did you adapt your practice to various waves of the pandemic? How did you adapt to changing regulations?
  4. What did you learn from these early changes and how did that change how you practiced going forward?
  5. How did your team support each other during this time?
  6. What were some of the biggest challenges?
  7. What were some of the biggest successes?

Section 3: Impact on patient population

1. What is your impression of how the pandemic has affected your patients?

Probing questions:

- 1. What has been the impact on their day-to-day lives?
  2. What has been the impact on their care?
  3. What existing challenges has the pandemic deepened?
  4. Have there been any new/unique challenges?
  5. Have there been any unexpected positive impacts?
  6. How did the changes you implemented affect your patients?
  7. Which changes/programs were the most impactful?
  8. What did you learn from this experience that may help improve the provision of palliative care to vulnerably housed patients in the future?

4. Final Thoughts

**APPENDIX 2: Coding Scheme**

| **Theme 1: FACTORS NEGATIVELY IMPACTING PATIENT HEALTH** | | | | |
| --- | --- | --- | --- | --- |
| Micro level | Patient risk factors | Health | Substance use   - Substance use as symptom management | 0011 - He [patient] has a substance use disorder – IV fentanyl use – and as a result of that he’s had a lot of structural heart infections. Like I don’t know, I think he had endocarditis but he doesn’t anymore, but now one of his valves needs to be replaced. And all as a result of his IV drug use. (Substance use as a risk factor)  0011 - And we know that when you’re purchasing substances because you know, possibly that is the medication that you need to medicate from the trauma and stigma and the pain and the life limiting illness diagnosis that you’re also battling. (Substance use as symptom management)  0016 - Now they’re in a room, not allowed out, not allowed to socialize literally in this room without anyone checking in on them, you know if you’re not using with somebody you can’t really administer your NARCAN if you’re overdosing so we saw a lot of overdose deaths- way too many. |
|  |  |  | Medical complexity | 0011 - I’m just thinking of one other person who has end-stage AIDS now. I think the CD4 count is like 50. And the person’s so resilient, and has bad Kaposi Sarcoma wounds everywhere and is a [unintelligible] and hasn’t left the house in 2 years.  0015 - patients who had metastatic cancer that were receiving chemotherapy or immunotherapy that put them at higher risk for contracting or serious complications of COVID-19. |
|  |  |  | Increased risk for patients receiving palliative care | 0015 - patients who had metastatic cancer that were receiving chemotherapy or immunotherapy that put them at higher risk for contracting or serious complications of COVID-19 |
|  |  | Housing | Homelessness | 0015 - A lot of us were concerned that individuals especially those living in shelters and those more transiently housed who were at higher risk of contracting COVID and dying because of high morbidity and mortality within the homeless populations. |
|  |  |  | COVID transmission risk in congregate settings | 0012 - in congregate housing it was like [COVID-19] could hit all of our patients and some could even die of COVID in these places. |
|  |  |  | Limitations of physical spaces | 0013 - there’s kind of a rush hour for the elevators and if one of the elevators goes down you’re like either going to risk getting into a crowded elevator at like 3 pm because one of the other elevators is down and it’s like literally one of the only elevators servicing the building. |
|  |  |  | Lack of fixed address | 0010 - having an address was a big part in getting home care involved which was frustrating at times and didn’t provide a lot of flexibility to provide care in a way that worked for the individuals |
|  |  | Trauma | Mistrust of medical system | 0010 - it helped us to bring this topic up again not only from a standpoint of the forgotten people who aren’t accessing care but like why aren’t they accessing care and what is it that happens if they go to emergency or are in the hospital that is so bad that they don’t want to |
|  |  | Food insecurity |  | 0012 - The simple things like people were not getting food and couldn’t go to the places where food is given out because they weren’t giving out food for a while |
|  |  | Lack of social support |  | 0011 - I think, you know, now that hospitals are saying that you have to have an essential care partner or you are only allowed to have one essential care partner, maybe the rest of us are thinking well you have 5 siblings, how do you only pick one. You have our patients who don’t have anyone. |
|  |  | Intersecting vulnerabilities |  | 0014 - We work with people who experience structural vulnerabilities including homelessness and poverty, but not limited to people who experience mental illness, who use drugs, who have had traumatic experiences with healthcare, people who have fallen victim to system racism, people who are over-policed, people with disabilities, people who are food insecure. |
|  | Barriers from providers | Stigma against the structurally vulnerable population |  | 0010 - And the stigma too. We recently had an outbreak of COVID in this wave in our inner city populations and so, that gets applied as a blanket to any of our clients |
|  |  | Multiple appointments |  | 0014 - Well what happens when you have a serious medical illness? You end up going to the hospital a lot – whether it’s for your medical appointments, for chemotherapy or dialysis, or for your surgeries, or because, you know, your… or even for palliative care sometimes. |
|  |  | Provider over-focus on COVID |  | 0016 - It seemed that legit health concerns weren’t considered- a lot were swabbed for COVID and then sent home. It was like no we sent him in there because we think he’s having a COPD exacerbation and double vision for someone we think is having brain metastases we need imaging and I would say 90% of people were just swabbed for COVID and then sent back home and that was frustrating because a lot of our clients don’t want to go to hospital - it’s a last resort so if we can gain enough trust to get them to go – only to get swabbed and then turned away. |
|  | Outcomes for patients during COVID | Medical | Increase in overdose deaths | 0016 - When they were reducing the number of people in shelters and placing people in the hotel programs, now they’re in a room, not allowed out, not allowed to socialize literally in this room without anyone checking in on them, you know if you’re not using with somebody you can’t really administer your NARCAN if you’re overdosing so we saw a lot of overdose deaths- way too many. |
|  |  |  | Increased severity of palliative issues | 0014 - we saw that the palliative care severity of issues really dramatically increased as compared to previous. |
|  |  |  | Worsening mental health | 0016 - One thing I specifically remember was seeing our clients who suffer from mental health disorders really start to spiral out of control – we saw mental health becoming much, much worse, people just losing their shit to be honest and it was because all the supports they had weren’t coming around anymore like case works or manager coming in daily to give meds and check in and all of a sudden they were totally alone – we saw a lot of mental health spiral out of control. |
|  |  |  | Frequent loss to follow-up | 0011 - Transportation to get to appointments is a big thing because I mentioned people were often lost to follow up. |
|  |  | Social | Displacement | 0015 - I think similarly to how I mentioned a lot of people being taken out of communities to go to shelter hotels, a lot of people suffered a lot more social isolation than they might normally. I think we see a lot of that with our clients who get housing through a terminal illness priority pathway and then they’re taken away from their communities and their main supports and family so we were seeing that in a more concentrated way because so many people were moved around from isolation centres to hospital or back to their shelter hotels and so I think a lot of our clients suffered a lot more social isolation than they might otherwise. |
| Meso level | Barriers | Health institutions | System is difficult to navigate | 0013 - we had to try to coordinate a transfer between two larger kind of acute care hospitals and then with one of the palliative care units in Toronto. And I remember going to those Zoom meetings and thinking that it was amazing kind of how challenging it was to work within the hospital system. |
|  |  |  | Inflexible institutional rules | 0011 - Palliative care units have such strict eligibility criteria. Like you can’t be a drug user and you can’t, you know, have mental illness, there can’t be any suicidality, there can’t be… all these things. And so that will also prevent people from accessing palliative care. |
|  |  |  | Limited access to visitors in hospital | 0013 - You had lots of people who were choosing not to receive care in hospice or PCU until it was absolutely necessary and I think that was… you know for someone who is living in the community and had their street family that was living with them and helping to support them knowing that they wouldn’t be able to come visit them or be with them at like one of the most vulnerable points in their life. I think many people were choosing not to pursue that. |
|  |  |  | Lack of recognition of chosen family | 0010 - and how do we get their chosen family recognized as chosen family, which is a barrier we have regardless of COVID or not. |
|  |  | Community resources | Shutting down of in-person resources during COVID   - Lack of access to community programs - Lack of access to safe opioid supplies - Lack of access to outreach services | 0016 - I would say it comes down to lack of resources. A lot of our clients would go to these drop-in centres to shower, to use the phone, to get a meal, to get clothes, to use the washroom and with all those services shut down people couldn’t take care of them hygienically. They couldn’t have a change of clothes, they couldn’t do their laundry, and they couldn’t use the washroom, so they just became stagnant and had to rely on what they had with them to survive through that whole period. (Lack of access to community programs)  0013 - to be honest I think it’s a reflection of the toxic street drug supply and lack of access to safe opioid supply programs, and harm reduction programs that would help support them and ensure that they knew kind of the drugs that they were using. I think that there’s… you know people who were dying from overdoses that I don’t think were intentionally overdosing. I think they were using drugs that they weren’t sure what was in them. (Lack of access to safe opioid supplies)  0014 - And there aren’t many community care teams like ours that exist in the sense that there are some of course but there’s not enough (Lack of access to outreach services) |
| Macro level | Barriers | Government | Gaps in government regulation | 0014 - We saw anytime there was an impact or a social policy that was being debated provincially or nationally, our population faced it first. So, you know, it just blew my mind that we had to advocate for people facing homelessness to be prioritized for Phase I vaccination, right? |
|  |  |  | Difficulty accessing government services | 0011 - You know, even things like getting a health card renewed is the kind of thing that during the pandemic, you know, Service [province] is saying “you have to have an appointment. You have to book it online.” It’s like but some people don’t have access to computer to book things online. |
|  |  |  | Lack of focus on prevention | 0016 - I think the funding also reflected that so organizations got ‘x’ amount of dollars to try and prevent outbreaks in congregate settings and in setting up the hotel programs and such but then the big funding came when the outbreak started happening and all of a sudden there were millions of dollars being funneled to treat rather than prevent. |
|  |  |  | Lack of recognition of needs of structurally vulnerable populations | 0015 - feeling like something devastating was going to happen to the population we serve and feeling we had little control over how we could prevent that because of a lack of government response to protecting the populations who were at highest risk. |
|  |  |  | Criminalization of drug use | 0013 - You know trying to deal with like substance use issues in a society that doesn’t value people who use drugs and criminalizes them I think is really what leads to these problems. |
|  |  |  | Restrictions on benefits | 0011 - when we’re working with someone who’s over the age of 65 who is now on pensions, especially if they had been on [provincial disability funding], they get cut off after that age. I don’t actually think I would have an answer for what I would do for people that don’t have the funds to afford a taxi trip to appointments when that service is cut off, or be able to afford a walker. |

| **Theme 2: USE OF TECHNOLOGY** | | |  |
| --- | --- | --- | --- |
| Barriers for Use | Lack of technology |  | 0014 - So how could we do some of the care virtually? Virtual care was a real luxury for people who work in mainstream health care or who have access to phones and internet and Zoom and things like that, but many of our clients didn’t have access to that.  0010 - Communication- connecting with the clients is probably the number one thing like how do we continue providing care when we’re being told to isolate and go remote to this population like we just don’t have the same access to technology that a large number of population have access to and I think it was easier for people with resources to pivot and harder for our clientele without access to that. |
| Facilitators for Use | Human support of virtual care |  | 0012 - We were actually just deploying tablets that people could come up and press a button and be in a waiting room to be seen by their remote team. We were giving out phones and tablets quite a bit and sometimes that was a platform like zoom or something else, sometimes it was just audio that could be used for that person or what they preferred.  0016 - We would facilitate phone visits for other providers during our visits. |
| Outcomes | Negative | Loss of in-person connection | 0010 - So we had to get a means of connecting with them and that went from face to face to phone alone and that’s such a barrier not only for actual connection but also for building a relationship and assessing. |
|  |  | Increase in virtual-only care | 0015 - I think a lot of people still follow-up with their primary care doctors- we share clients- and also a lot of them have specialists because a lot of them are navigating advanced and chronic illnesses and so it was hard for a lot of them to access those supports because of the virtual nature that a lot of clinicians shifted towards. |
|  | Positive | New connection opportunities | 0016 - I’d say one of the biggest successes was specialists being willing to speak with clients not in person. So for me to get client A who hates hospitals, hates docs, hates waiting 3 hour for an appointment into a hospital to wait for a 15 minute visit with a specialist who basically told them nothing- sorry, happens- but now all of a sudden they’re ok we can do this by phone- we can do this by whatever works- now I can do it in the client’s space, where they feel comfortable, where they feel in control and now we’re not losing patients to follow up because they just don’t want to go to their appointment. |
|  |  | Care expansion | 0015 - [Virtual care] allowed us to see or follow up with more people in a day because our geographic area is so large that we could address issues with more patients if we were able to incorporate more virtual care into our practice.  0014 - I think another thing that we learned is that there probably is a role for virtual care in this work. It’s probably not the same as what we hear in conversations around mainstream palliative care because ultimately many of the people we care for don’t have access to technology. But when they did get access to technology it was actually ok and did work for many people. So we have a digital poverty or digital inequity that exists, but once the digital inequity is kind of solved, it’s kind of incredible what can happen with some of the people we care for. |

| **Theme 3: PROVIDER EMOTIONS** | | | |
| --- | --- | --- | --- |
| Increased emotional demands | Fear of COVID |  | 0010 - For a while, I would wake up in the morning and think, “Oh my god what if I’m called last minute to support a COVID death in community and what is that going to look like and am I putting my family at risk.” So it’s not just the stress of supporting someone who is dying there’s also the stress of I’ve never seen a death like this and so there’s that anticipatory stress of the day in and day out. |
|  | Grief | Overlapping grief | 0014 - There is a grieving community out there. We have got an opioid death crisis, we’ve got a homelessness crisis, and a COVID-19 crisis, and people are facing moral injury and compassion fatigue. |
|  |  | Impact of unexpected death | 0013 - I think the other thing with the death… like the overdose deaths is that they’re unexpected deaths unlike the deaths we see with our teams. I think there’s not a lot of time to prepare people, help prepare staff members. And it was interesting because I think the overdose crisis and the overdose deaths that we saw really impacted a lot of the service workers perception of death and dying. And the fact that they were seeing all of these unexpected deaths and how, you know, they were just expected to bounce back and that room would be filled with like another person the next day, type-of-thing. |
|  | Work Stressors | Risk of staff being off work | 0012 - with the pandemic starting we were trying to figure out if one of the doctors on the team needed to isolate or had tested positive for COVID, what the team would do about it. Because obviously if we lose a day of physician work it… for people who really acutely… who have acute palliative care needs things can happen pretty urgently. (Risk of staff being off work) |
|  |  |  |  |
|  |  |  |  |
|  |  |  |  |
|  |  |  |  |
|  |  |  |  |
|  |  |  |  |
|  |  | Increased pressure on outreach teams | 0014 - We saw more volumes referred our way from acute care facilities than ever before in terms of the raw number, they were referred earlier in the trajectory of their admissions, and they were often discharged with a lot of complexity associated with them that would previously have been addressed in the hospital but were now… we were being asked to address them because the hospital was facing so much pressure due to COVID-19 admissions and ICU admissions and things like that.  0011 - I mean I think in general in the [team] we received so many more referrals during the pandemic as compared to before from what I understand compared to before. And so you think about the pandemic people lose their jobs and then people aren’t able to pay the rent and the bills and all these things. And you find people who lose their housing and become homeless or are couch surfing and all of that.  0014 - More of the social service supports were required by community care teams like us. And there aren’t many community care teams like ours that exist in the sense that there are some of course but there’s not enough… there was not enough to meet the demand and need int that way. |
|  |  | Loss of team connectivity | 0016 - It was really hard because were such a social team and prior to the pandemic, we would go out all the time, have work gatherings all the time, and we used that as a therapeutic approach to debriefing since the last time we got together and talking about difficult cases and supporting each other through that so we really had to shift a lot of our supportive measures for each other to these zoom meetings and it wasn’t easy. |
|  | Moral Distress |  | 0015 - Our initial reactions were being worried about the populations we serve. A lot of us were concerned that individuals especially those living in shelters and those more transiently housed who were at higher risk of contracting COVID and dying because of high morbidity and mortality within the homeless populations so there was definitely some moral distress and feeling like something devastating was going to happen to the population we serve and feeling we had little control over how we could prevent that because of a lack of government response to protecting the populations who were at highest risk. |
| Resilience factors | Comfort | Increased comfort with pandemic reality | 0011 - I think as the time has gone on, it sounds bad but I feel like I sort of have let my guard down in the sense that I just… I really see that I can’t let the pandemic be the reason why I can’t see someone or why I won’t go into a shelter space that has an outbreak. |
|  |  | Accommodation of different comfort levels of team members | 0013 - We tried to accommodate everyone’s comfort level so there were some physicians who preferred to do home visits while possible or there were physicians who felt they could do better care by providing in person so we kind of left it up to the physician’s discretion unless it was absolutely urgent for in-person like a crisis. We kind of left it to people’s comfort levels but people were seen one way or another. |
|  | Coping Strategies | Team debriefings | 0016 - if there’s a really complicated case, challenging case, just calling me and like ‘are you okay, how are you doing, how are you feeling about this’. |
|  |  | Collegial support | 0016 - We really had to shift a lot of our supportive measures for each other to these zoom meetings and it wasn’t easy. We’re a very in-person social team- but it was just a matter of making sure everyone felt supported, checking in on one another. |
|  |  | Allowing space for grieving | 0014 - we hold a minute of silence and we light a candle and we cry together, we laugh together, we tell stories about what it’s like to… you know remember what it was like to care for that person and reflect on what it was like to care for them, and think about ways that we can renew and re-invest in each other … And then we hold another minute of silence, blow the candle out, and then go out and do it again. It’s really become a ritual and really become a structured space for people to grieve with people that they know kind of get it and understand it because they were on the journey with them. |
|  |  | Self-care | 0010 - Creating that work life balance is a big part of sustaining ourselves and being able to – for some people, to walk into your office and your house and allow yourself to take care of life things on your break or go for a walk or be removed from that office dynamic. |
|  |  | Maintaining boundaries work hours | 0011 - I think that one thing that worked really well for me that just, you know, it’s not always good practice to be working after hours and at all hours of the day you know for your own personal self care. |
|  |  | Unhealthy coping strategies | 0010 - I know at the beginning of COVID I was probably drinking more which wasn’t helpful at all. |
|  | Resilience of individuals |  | 0012 - Trusting and realizing if we face this then we can face anything [with] the resilience of us as healthcare providers but also the resilience of patients and the people around them.” |

| **Theme 4: COLLABORATION WITH COMMUNITY AND HEALTH CARE ORGANIZATIONS** | | |
| --- | --- | --- |
| Interprofessional |  | 0011 - There are also other professionals that we work with interdisciplinarily to address the social and physical care needs of the clients. So that includes OT, PT, speech, and we are seeing PSW as well. |
| Organizations | Health institutions | 0010 - it may be a street outreach group that’s walking the streets and notices someone is living in a tent that notices that their health doesn’t seem too great and that they’re certainly at risk of dying so we’ve had referrals in that nature and will go out and have conversations with the individual and see how we might be able to support them with their goals of care and it may be a role of acting as a consultant to inpatient teams around someone who may have had substance use and addictions and may be requiring help with pain management the context of a palliative diagnosis and so we can sort of be taking more of a consult based role where we never actually meet the individual but we offer supports by phone or suggestions in terms of approach to actual hands on care delivery when no other clinical team is involved. |
|  | Community organizations | 0014 - But then I would say we have other organizations and relationships that make the work happen external to the direct [team]. This includes various respite shelters, drop-ins, housing agencies, mental health agencies, case management organizations. So sort of I would say the homelessness and mental health sector and community. |
|  | Charity organizations | 0011 - so we also work with um Hospice Toronto. And they provide volunteers that go in to do caregiver respite and provide help with meal prep and things like that. So it’s multi-volunteer. It’s not volunteer run but like volunteer driven and then they have like case managers. |
| Patients |  | 0012 - then we have clients/patients for other things who say ‘oh my goodness this has been really helpful for me, I also know someone who is struggling with advanced liver failure can you see them?’ so we also have patients referring patients. |
| Community |  | 0016 - I think people in general have just become nicer to one another- it gave us a solid front to fight- not everybody obviously, but it brought a lot of people together working towards common goals. Especially in homeless health a lot of people came together to come up with creative strategies and problem solving to make things work and it brought a lot of people closer together. Our team became closer because we had to get pretty damn creative with a lot of clients in figuring out how we’re going to make things work to make sure they felt supported. It did bring a lot of people together – it gave us a common enemy: COVID. |

| **Theme 5: CARE PROVIDER EDUCATION AND ADVOCACY** | | |  |
| --- | --- | --- | --- |
| Micro level advocacy | Patient | Lack of ability to self-advocate | 0011 - But the one about going to the hospital was a big one for me because it just made me feel so sad that… I’m sure that if they didn’t have some kind of advocate with them that they would just end up walking away and not coming back. |
|  |  | Healthcare worker filling caregiver role | 0011 - So I guess when it comes to the pandemic I’m just thinking about the fact that I’m in the role of an essential care partner and just to honor that and to always be respectful and advocate and speak up with that person kind of like alongside me so I can advocate for them while using their voice as much as possible. |
| Meso level advocacy | Advocating to shelters |  | 0015 - Sometimes it would be talking to the shelter about giving people more space so trying to get them out of shared room and into a room where they had more personal space and less crowding, and other times we built relationships with the shelter hotels that were starting to develop and were able to get some of our patients transferred out of shelters and into shelter hotels where they would be living in their own individual rooms. |
|  | Advocating to healthcare institutions |  | 0014 - And worked within [larger inner city health organization] to work on their model to address COVID-19 through the recovery models which ended up being the shelter hotels that you’ve heard a lot about.  0011 - this person had stage four lung cancer and was in so much pain and basically he needed to go into [hospital] for tons of appointments and things like that, and they were looking at different types of approaches to treatment and whatnot. His pain was so bad that we advocated with [MD] and his medical oncology and radiation oncology [teams] at [hospital] to get him an inpatient stay for 6 weeks where they did all his chemotherapy, his radiation, and then he was just there for pain management. |
|  | Expansion of community resources |  | 0014 - We were holding 2-3 grief circles a week, actually, as a team and so, you know, here we go, this palliative care team kind of becomes kind of like a grief response. And the grief intervention was a huge part of what we did for the overall COVID homeless response in [city]. |
| Macro level advocacy | Advocating to governments |  | 0014 - So, you know, it just blew my mind that we had to advocate for people facing homelessness to be prioritized for Phase I vaccination, right? |
|  | Media | Creating public presence | 0014 - We continued to use our narratives from the streets to drive advocacy through media, through op-ed writing, through a public presence. You know, the public knew we were doing this work during COVID-19 amidst the crisis and we were able to leverage that to showcase the importance around ending inequities like homelessness and addressing social inequities. |
|  |  | Empathy as advocacy tool | 0014 - We leveraged the voices of people with lived experience to give truth to power to their voices by working with media and doing public stories during the pandemic to showcase the plight of people experiencing homelessness so that we did get more empathy and compassion from Canadians. |
| Leading change |  |  | 0014 - I think that we recognize the importance of being part of the advocacy and the leadership work that goes on in addressing homelessness and supporting homeless populations. |
| Teaching others | Expanding competency in palliative care |  | 0013 - I think advanced care planning is a topic that is challenging for lots of non-palliative care providers to explore but even more so for people who don’t have a healthcare background. So we had put together a presentation around that and some kind of practical guides for the [other medical team] on how to document who would be someone’s substitute decision maker or if they wanted to have further conversations with like our team or their primary care provider or team about advanced care planning. |
|  | Expanding competency in serving vulnerable populations |  | 0013 - the hospice actually came to us looking for guidance around how they could help service people who are using substances in the future and how our teams could collaborate more to provide more person-centered care for people who use drugs. And ultimately I ended up giving a talk to the hospice team which was really… started some really good discussions. |
| Knowledge gaps | About COVID |  | 0014 - Yeah I think that one of the first things that really came about was trying to learn about the virus and how this was going to have a direct impact on community-based health resources, which we are one of. |
|  | About palliative care for external healthcare provider |  | 0014 - we really recognized early on in the palliative care journey that many of the people who work on the frontlines of this homelessness and palliative care intersection, our social care workers and health workers, would typically not get a lot of support and even training around how to address grief and bereavement. |
| COVID learning | Improvement through scholarship |  | 0014 - We did our best to do was to keep track of the science at a general level around COVID, but also keep track of academic scholars who were doing work in homelessness and COVID around the world. I mean there would be new papers that came out about the ways that COVID was spreading in congregate settings like homeless shelters, for example. And that gave us guidance and understanding and we ended up actually, you know, writing… doing some of that work ourselves. |
|  | Learning from other teams |  | 0015 - We have an app that we chat through and were having regular meetings and informal discussions about how we were individually practicing and what we were seeing in our other workplaces and how we could adapt that to our [team] practice. |
|  | Decreased students and trainees |  | 0016 - I guess we also weren’t having learners in our vehicles anymore but a lot of the learners were suspended |
|  | Pandemic increasing visibility of inequities |  | 0012 - I think having COVID allowed us to sharpen that lens and focus that light in a way that people can understand in a way that ‘oh yeah it was hard for me to get my groceries,’ oh yeah it was hard for me to get my medicines’, oh yeah I couldn’t get to the hospital well and yeah that was hard for you think about how hard it is if you don’t have someone to support you, think about whether you don’t have a home or think if you don’t have money so I think coming back to another question but I think it just allowed more people to recognize how vulnerable people can be and how our systems aren’t normally created to support all people. |

| **Theme 6: OUTREACH TEAM FACTORS** | | |  |
| --- | --- | --- | --- |
| Outreach model benefits | Flexibility |  | 0010 - Being an outreach team, we are used to working in a flexible setting and that will always be the case. I think that’s one thing about the work that’s really great- we are very autonomous and we are given free reign.  0013 - I mean I think of all of them, like, the idea of adaptability is like something that our team is most known for. |
|  | Addresses needs of structurally vulnerable populations | Leveraging financial resources | 0011 - So I just think about, you know, for anyone on [provincial disability funding] I would connect with the case workers and get them set up with a taxi chit that [is covered] so we can get them to and from all medical appointments. Sometimes I would accompany them in the taxi or drive behind the taxi and then drive back and make sure that they’re home safe. |
|  |  | Increasing permanent housing | 0011 - You’re working as a team to coordinate care and I think it’s a success story because in the end we were able to… [MD] signed one of those applications for housing, so he was able to get offered housing in an area that he really loves, and he’s still there. |
|  |  | Personalizing the approach to care | 0011 - And I think one of the things that I love so much about [team] is that it’s so flexible and not just [unintelligible]. Like you’re doing your drugs, you’re drinking, and you’re doing what you need to do, and you’re using with people. Yes? Ok good. And so you’re able to implement that harm reduction and trauma informed care in the work that you do.  0013 - I think it’s [COVID-19] really highlighted how important our model of care is for people. Because I think a population that didn’t have access to virtual care needed an outreach support model, and needed a team that could kind of meet them where they’re at and see people longer. |
|  |  | Patient autonomy | 0010 - it’s his choice and people can make that choice if they wish and I think it’s really great because it’s like informed decision-making – that’s palliative care like you can choose to not go and we aren’t going to give you a hard time about that. |
|  |  | Seeing clients in their own space | 0015 - in a city like [city] where there are so many palliative care supports, a lot of people facing barriers to care really require someone meeting them where they’re at – meaning physically going to where they are. |
|  | Model resilience |  | 0014 - I think what it really did show is, you know, this team is… and this model of care… you know, albeit very mobile and agile, is actually quite resilient and was resilient to the stresses of a pandemic. That says a lot about the care model and the ability to deliver a mobile distributed care. Because there were many nights where I would sit up and think, you know “do we really have what it takes to be resilient to the stresses of what the pandemic is bringing upon us?” |
|  | Broad scope of practice | Work across multiple settings | 0016 - Pretty much anywhere- in the street , in encampments, in a park where they stay, sometimes in coffee shops, sometimes in random places. We also do shelter settings so congregate shelters, hotel shelter programs and shelters specifically for domestic violence or refugees and newcomers to Canada. We are also in precarious or vulnerably housed individuals- so risk for eviction, unstable housing, housed but not stably, people who couch surf or stay with friends and family. Also some individuals who live in abandoned buildings- we do some of that as well so a little bit everywhere. (Work across multiple settings) |
|  |  | Coverage of large area | 0011 - we see people from all across the [municipal area] so we’re not just focused in the downtown core. We do go to [other city], we go to [other city], we see the downtown core, and we go as far east as [other city]. |
|  |  | Large variation in patient demographics | 0012 - Definitely structurally vulnerable, vulnerably-housed, issues with substance use, maybe mental health issues- these are people who sometimes have diagnosis of cancer, but not all times, some have advancing organ failure or at risk for that- so liver failure, COPD and often because we’re taking referrals from housing providers from no medical background, these people just look unwell and maybe have not had connection with service for a while so we’ll end up going out to see somebody in their tent and do a little bit of inquiry and then determining what it is they need within their health within their goals. We get referrals with people who have obvious EOL diagnoses but others that are more undifferentiated. |
|  |  | Combining models of practice | 0010 - it may be a street outreach group that’s walking the streets and notices someone is living in a tent that notices that their health doesn’t seem too great and that they’re certainly at risk of dying so we’ve had referrals in that nature and will go out and have conversations with the individual and see how we might be able to support them with their goals of care and it may be a role of acting as a consultant to inpatient teams around someone who may have had substance use and addictions and may be requiring help with pain management the context of a palliative diagnosis. |
|  |  | Using broad definition of homelessness | 0011 - Even though people are housed I still think it’s precarious housing and it’s vulnerable… you know they are vulnerable. For people of course who are couch surfing certainly that situation, that living situation where they’re in a, say, an apartment it’s typically very precarious. |
|  | Team composition | Interdisciplinary care | 0012 - So … we have a team of physicians, nurse outreach worker, and social workers some of them, most of them being part time physicians working with the structurally vulnerable in our community. |
|  |  | Diversifying team roles | 0014 - We didn’t need like five more palliative care physicians, we needed [social worker]. And that’s… in other words we grew but we grew in a way that met the needs of people where they’re at. What we needed was social care work that really had expertise in palliative care and homelessness together. And that was really crucial. We needed psychiatry and we needed mobile psychiatry, and that’s what [psychiatrist] had to offer. |
| Teamwork facilitators | Shared team values |  | 0011 - I was able to fit in with the team because I think our values align and I think that’s really important when you’re working on a team. |
|  | Lack of hierarchy |  | 0016 - I’d say it’s definitely the best team I’ve ever worked on. As a nurse you see a lot of doctor-nurse hierarchy, there’s none of that in this team, everyone is considered equal and there are no decisions made without the input of everyone being considered. Yeah I love it. |
| Adapting to pandemic | Safety | Keeping team members safe | 0014 - I’m also super proud that we were able to keep people safe. You know, these are the kinds of things in a pandemic that like, as a leader, that I think about. You know none of our colleagues got sick. Nobody to my knowledge got COVID-19; nobody ended up in hospital. You know like raw realities… I work on some other teams in other roles with other hats where that did happen and that’s really sad. So I think safety is paramount. |
|  |  | Access to PPE | 0013 - I think the big thing was getting access to PPE. I think we got a big donation of masks. We got… we were supported through [other medical team] I believe in terms of supplying gowns, N95s, face shields, those types of things. |
|  |  | Balancing safety and connection | 0015 - so there was a lot of discussion about how we could adjust our model to include more virtual care in a population that already faces a lot of barriers and to try and balance that without exacerbating the barriers they already face to care. |
|  | Adapting work to new regulations | Maintaining in-person care | 0016 - I think not grounding our team- that was the biggest impact we had. We were one of the few teams saying like we aren’t just going to ground people like people need us and that’s what we’re here for. So I think we developed a lot of trust with our clients and a lot of trust with some of our partner agencies or agencies we collaborate with that we weren’t going to give up on anyone- that we were going to go out and meet people even if it is in hazmat suits and we look ridiculous, but we weren’t going to give up on anyone.  0014 - We learned that for a certain segment of the people we care for, people with serious mental illnesses, for people who have had… who are very traumatized for example, there’s no technology that would ever work. So there’s always that sub-population that we need that in-person support and care. |
|  |  | Providing support for isolation needs | 0010 - and then it was really about supporting caregivers and clients with isolation so that they could stay safe. |
|  | Triaging patients |  | 0015 - We only pivoted to virtual care for clients that we felt could manage it- those who could reliably answer their phone and others we continued to see in person. |
