# Supplementary material for: Exploring the Barriers and Facilitators Experienced by Palliative Health Care Providers Working with Patients Experiencing Homelessness during the COVID-19 Pandemic
Source: Palliat Med Rep. 2023 Jan 17;4(1):3–8. doi: 10.1089/pmr.2022.0051 (PMC9892914; doi:10.1089/pmr.2022.0051)
Supplement: Supplemental data [file Suppl_TableS1.docx]

Table 1. Themes

| Theme | Quote |
| --- | --- |
| Theme 1: Factors negatively impacting patient health | “In congregate housing it was like [COVID-19] could hit all of our patients and some could even die of COVID in these places.” (Participant 0012) |
|  | “I would say it comes down to lack of resources. A lot of our clients would go to these drop-in centers to shower, to use the phone, to get a meal, to get clothes, to use the washroom, and with all those services shut down, people couldn’t take care of themselves hygienically.” (Participant 0016) |
| Theme 2: Use of Technology | “Virtual care was a real luxury for people who work in mainstream health care or who have access to phones and internet and Zoom…but many of our clients didn’t have access to that.” (Participant 0014) |
|  | “There was a lot of discussion about how we could adjust our model to include more virtual care in a population that already faces a lot of barriers and to try and balance that without exacerbating the barriers they already face to care.” (Participant 0015) |
|  | “I’d say one of the biggest successes was specialists being willing to speak with clients not in person…now I can do it in the client’s space, where they feel comfortable, where they feel in control.” (Participant 0016) |
| Theme 3: Provider Emotions | “For a while, I would wake up in the morning and think, “Oh my god what if I’m called last minute to support a COVID death in the community and what is that going to look like and am I putting my family at risk.” So it’s not just the stress of supporting someone who is dying; there’s also the stress of I’ve never seen a death like this.” (Participant 0010) |
|  | “If we don’t address the grief of our frontline workers, both in social care and in healthcare, then we will never be able address or meet the needs of people experiencing homelessness in Canada. There is a grieving community out there. We have an opioid death crisis; we’ve got a homelessness crisis and a COVID-19 crisis, and people are facing moral injury and compassion fatigue.” (Participant 0014) |
|  | “We really had to shift a lot of our supportive measures for each other to these Zoom meetings, and it wasn’t easy. We’re a very in-person social team. But it was just a matter of making sure everyone felt supported, checking in on one another.” (Participant 0016) |
| Theme 4: Care Provider Education and Advocacy | “I think advanced care planning is a topic that is challenging for lots of non-palliative care providers to explore but even more so for people who don’t have a healthcare background. So we had put together a presentation around that and some kind of practical guides for the [other professionals] on how to document who would be someone’s substitute decision maker.” (Participant 0013) |
|  | “We leveraged the voices of people with lived experience to give truth-to-power to their voices by working with media and doing public stories during the pandemic to showcase the plight of people experiencing homelessness, so that we did get more empathy and compassion from Canadians.” (Participant 0014) |
| Theme 5: Outreach Team Factors | “I think [COVID-19] has really highlighted how important our model of care is for people. Because I think a population that didn’t have access to virtual care needed an outreach support model and needed a team that could kind of meet them where they’re at and see people longer.” (Participant 0013) |
|  | “I think not grounding our team was the biggest impact we had…we developed a lot of trust with our clients and a lot of trust with some of our partner agencies or agencies we collaborate with that we weren’t going to give up on anyone.” (Participant 0016) |
|  | “I think what it really did show is, you know, this team is… and this model of care… you know, albeit very mobile and agile, is actually quite resilient and was resilient to the stresses of a pandemic. That says a lot about the care model and the ability to deliver mobile, distributed care.” (Participant 0014) |
